# Nitrogen-Doped Carbon Dots from *Averrhoa carambola* Fruit Extract as a Fluorescent Probe for Methyl Orange

**DOI:** 10.3390/s19225008

**Published:** 2019-11-16

**Authors:** Muhammad Zulfajri, Sandhiya Dayalan, Wang-Yu Li, Chia-Jung Chang, Yuan-Pin Chang, Genin Gary Huang

**Affiliations:** 1 Department of Medicinal and Applied Chemistry, Kaohsiung Medical University, Kaohsiung 80708, Taiwan; 2 Department of Chemistry Education, Universitas Serambi Mekkah, Aceh 23245, Indonesia; 3 Department of Chemistry, National Sun Yat-sen University, Kaohsiung 80424, Taiwan; 4 Department of Medical Research, Kaohsiung Medical University Hospital, Kaohsiung 80708, Taiwan

**Keywords:** *Averrhoa carambola*, fruit extract, L-arginine, amino acid, nitrogen-doped carbon dots, hydrothermal, methyl orange, fluorescent probe

## Abstract

In this study, a simple and green hydrothermal treatment was performed to prepare nitrogen-doped carbon dots (NCDs) from *Averrhoa carambola* (AC) fruit extract as a carbon precursor and L-arginine (Arg) as a nitrogen dopant. The AC-NCDs were characterized by UV light, fluorescence spectroscopy, transmission electron microscopy, FTIR spectroscopy, Raman spectroscopy, UV-vis spectroscopy, and zeta potential analyzer. The AC-NCDs were spherical and the average diameter was estimated to be 6.67 nm. The AC-NCDs exhibited the maximum emission intensity at 446 nm with 360 nm excitation wavelength. The fluorescence quenching behavior of AC-NCDs after interacting with methyl orange (MO) dye was studied. The interaction of AC-NCDs and MO was achieved within 3 min and the fluorescence quenching was maintained to a fixed value even after 30 min. The linearity was obtained in the range of 1 to 25 μM MO with a 0.30 μM detection limit. Furthermore, the pH values affected the quenching behavior of the AC-NCDs/MO system where the interaction mechanisms were driven by the electrostatic interaction, π–π interaction, inner filter effect, and energy transfer. The pH 5 maintained higher quenching efficiency while other pH values slightly decreased the quenching efficiency. Incoming applications, the AC-NCDs can be used in various important fields, especially for environmental protection.

## 1. Introduction

Recently, carbon dots (CDs) exhibiting strong fluorescent properties with excitation-dependent emissions have arrested much attention [1]. CDs are superior in their unique properties such as good water solubility, low toxicity, high photostability, good biocompatibility, excellent optical properties, good electrical conductivity, and easy functionalization or modification [2]. Several parameters, including reaction time, reaction temperature, particle size, dopant nature, carbon precursor amount, pH value, and solvent present a key role in increasing fluorescent properties of CDs which can be utilized as the chemical sensor, biosensor, bio-imaging, catalyst, drug carrier, and energy storage device [3,4,5]. To increase the fluorescent properties, the dopants of boron (B), nitrogen (N), sulfur (S), and phosphor (P) were commonly added to functionalize CDs [4]. Among these heteroatoms, N-dopant is a widely used dopant to increase the fluorescent properties of CDs.

Nowadays, the chemistry concepts of economical and green materials attract much attention to the synthesis of CDs. Cheap and available natural resources are still challengeable as the carbon precursors to produce CDs [6]. Various top-down and bottom-up synthesis methods have been promoted to produce CDs including hydrothermal, microwave, ultrasonic, laser ablation, electrochemical oxidation, and chemical oxidation treatments [7]. However, the hydrothermal treatment is widely used as a green, straightforward, affordable and soft chemical route method by using natural resources for the production of CDs which results in uniform particle sizes [8,9]. *Averrhoa carambola* (AC), one of the natural resources, is often known as “star fruit” from the family of *Oxalidaceae* and widely planted in India, China, and Southeast Asia [10,11]. AC fruit is globally marketed due to its pharmacological significances including analgesic, hypotensive, hypocholesterolemic, hypolipidemic, antitumor antimicrobial, antifungal, anti-ulcer, and antioxidant activities [11]. The main constituents in the crude AC fruit extract are saponins, alkaloids, flavonoids, tannins, proanthocyanidins, epicatechin, gallic acid, L-ascorbic acid, sterols, fatty acids, sugars, minerals, volatile favors, dietary fibers, pectin, cellulose, hemicellulose, carotenoid, carbohydrates, proteins, calories, fats, carotene, tartaric acid, oxalic acid, α-ketoglutaric acid, and citric acid [12]. In this study, the AC fruit extract has been chosen as a carbon precursor for the production of CDs. Besides, amino acids can be used to produce heteroatom-doped CDs with increased fluorescent properties that undergo dehydration and decarboxylation reactions in the synthesis process [13]. L-arginine (Arg), an amino acid, has been chosen as N-dopant to react with the primary carbon precursor (AC fruit extract) which has N content approximately 32% [14].

**Figure 1:**
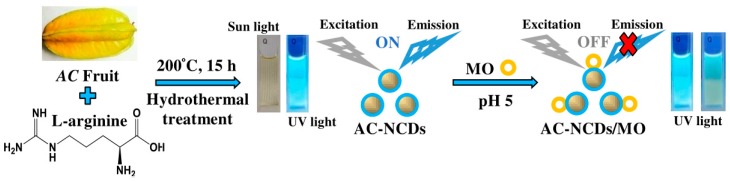
A schematic representation of *Averrhoa carambola* (AC)-nitrogen-doped carbon dots (NCDs) synthesis and application as a fluorescent probe for methyl orange (MO).

On the other hand, organic dyes (ODs) are widely used in food, pharmaceutical, plastic, cosmetic, paper, rubber, textile, and leather industries [15]. The leftover of ODs is considered a wide variety of pollutants in the natural water resources or wastewater treatment systems. Their release into water can render environmental pollution because the ODs are commonly toxic and carcinogenic for human health [16]. Thus, the probing of ODs is of high significance. Methyl orange (MO), one of the ODs, is a variety of p-aminoazobenzene dyes and widely used in the textile industry and chemical laboratory as an acid-base indicator [17]. But, the MO solution is toxic and irritating for human. So far, there are very few reports about the detection methods of MO [18,19,20,21]. Therefore, it is needed to promote facile, simple, and efficient methods. Toward this objective, a facile and green hydrothermal treatment was adopted to synthesize AC-nitrogen-doped carbon dots (AC-NCDs) from AC fruit extract and Arg. The physicochemical and optical properties of AC-NCDs were characterized. The fluorescence behavior of AC-NCDs after interacting with MO was also evaluated. Thus, AC-NCDs can be used as a higher selective and sensitive fluorescent probe to detect MO. The schematic representation of the synthesis and application of AC-NCDs as a fluorescent probe for MO was demonstrated in Figure 1. Comparing to the direct measurements by UV-vis spectrometry or fluorescence spectrophotometer, the proposed AC-NCDs fluorescent probe for MO provides the advantage of better selectivity and free from several spectral interferences. As far as we know, there is no other report on the production of NCDs utilizing AC fruit extract with Arg. The use of other NCDs as fluorescent probes for MO detection has also not been declared.

## 2. Materials and Methods

### 2.1. Materials

AC fruit was purchased from a 24h supermarket in Sanmin District, Kaohsiung City, Taiwan. Arg, alizarin (AZ), eosin B (EB), crystal violet (CV), and rose bengal (RB) were obtained from Alfa Aesar (Ward Hill, MA, USA). MO and purpurin (PP) were obtained from Sigma-Aldrich (St. Louis, MO, USA) and TCL Chemicals (Tokyo, Japan), respectively. Methylene blue (MB) was purchased from Acros Organics (Geel, Belgium). NaOH and HCl were obtained from Showa Chemical Industry Co., Ltd (Tokyo, Japan) and J.T. Baker (Phillipsburg, NJ, USA), respectively. Citrate-phosphate buffer was prepared from disodium hydrogen phosphate and citric acid anhydrous obtained from Showa Chemical Industry Co., Ltd (Tokyo, Japan). The ultrapure water from the Simplicity^®^ Millipore Water Purification System (Burlington, VT, USA) was used as a solvent for all experiments.

### 2.2. Preparation of AC Fruit Extract and AC-NCDs

AC fruit was washed several times with ultrapure water, sliced into small pieces, and blended with a domestic blender. Subsequently, the crude extract was centrifuged (8000 rpm, 10 min) to separate the pulp residues. The upper layer suspension was taken as the AC fruit extract and kept at 4 °C in the freezer. Afterward, AC-NCDs were synthesized by the hydrothermal treatment method. In a typical procedure, the AC fruit extract was mixed with ultrapure water (25:75, %). 0.6 g Arg was added into 10 mL of the mixture and then transferred into hydrothermal equipment (200 °C, 15 h) after 2 min sonication. Furthermore, it was allowed to cool down naturally and filtered with a Whatman filter paper to separate large residues. The solution was further purified through centrifugation (9000 rpm, 30 min) followed by filtration with a Whatman filter paper and 0.2 μM PES syringe filter to dismiss residual large solid particles/starting materials and stored at 4 °C for experiments. To optimize the synthetic conditions, various solvent volume percentages, hydrothermal temperatures, hydrothermal reaction time durations, and Arg weights were evaluated.

### 2.3. Characterization of AC-NCDs

The surface morphology of AC-NCDs was characterized by using the transmission electron microscopy (TEM; Hitachi HT-7700 microscope). The functional groups of AC-NCDs were recorded by performing FTIR spectroscopy (ALPHA FTIR Spectrometer, Bruker). The UV-vis absorbance was measured by using Spectra Academy UV–Visible Spectrometer Detector SV-2100. Raman spectrum of AC-NCDs was measured by Micro Raman Identify Spectrometer from ProTrusTech Co. Ltd. (Tainan, Taiwan). The fluorescence spectra were monitored with a Varian Cary Eclipse Fluorescence Spectrophotometer. The zeta potential values were done by using a NanoPlus HD-Zeta/Nano Particle Analyzer.

### 2.4. Fluorescence Stability of AC-NCDs

The effect of pH values (pH 2–12) on the fluorescence behavior of AC-NCDs was adjusted with 0.1 mM HCl-NaOH solutions, whereas the effect of ionic strengths was measured after adding with various NaCl concentrations (0–1000 mM) to AC-NCDs’ solution. Besides, the light irradiation with different irradiation times (0–60 min), storage times (1–30 days), and heating temperatures (25–100 °C) of AC-NCDs were measured. In all cases, the fluorescence spectra were recorded with 360 nm excitation wavelength for comparison.

### 2.5. Measurement of Fluorescence Quantum Yield

In brief, 0.1 M sulfuric acid was used to dilute quinine as a reference (quantum yield (QY) = 54%, η = 1.33), while ultrapure water (η = 1.33) was used to dilute AC-NCDs. The absorbance value at 360 nm was kept below 0.1 and the fluorescence spectrum with 360 nm excitation was recorded. The integrated fluorescence intensity and absorption value of AC-NCDs were compared to the quinine solution by using a formula:

***QY*_A_** = ***QY*_B_ (*A*_B_/*A*_A_) (*I*_A_/*I*_B_) (*η*_A_^2^/*η*_B_^2^),**

where, the “***QY***” points to the quantum yield, “***A***” points to the absorbance, “***I***” points to integrated fluorescence intensity, and “***η***” points to the refractive index of solvents. The “**A**” and “**B**” subscripts point to AC-NCDs and quinine solution, respectively.

### 2.6. Fluorescent Probe for MO

The interaction between AC-NCDs and MO was measured at room temperature. For selective probe, AC-NCDs’ solution (60-fold, 900 µL, pH 5.0 with citrate-phosphate buffer) and each OD (100 µL, 50 μM) were sequentially added into 1500 µL Eppendorf tubes. We performed 3 min of interaction time (1 min of mixing time, 2 min of incubation time) before measuring with a fluorescence spectrophotometer. The fluorescence spectra were measured at 360 nm excitation wavelength. For the sensitive probe, various concentrations of MO solution (100 μL) were added into AC-NCDs’ solution (60-fold, 900 μL). The fluorescence behavior of the AC-NDs/MO system was recorded at 360 nm excitation wavelength. The fluorescence behavior of the AC-NCDs/MO system under different pH values was also measured.

### 2.7. Probing of MO in Tap Water Samples

The applicability of AC-NCDs as a fluorescent probe for MO in real water samples was also evaluated. The tap water was utilized without any filtration or purification. The samples were spiked with the standard MO solution and added to the AC-NCDs’ solution to a final concentration of 5–75 μM. The fluorescence spectra of the samples were measured. To obtain the recovery percentages, the below formula was used:

***R*** = **[(*C_c_* − *C_b_*)/*C_a_*] × 100%,**

where, “***R***” is the recovery percentage, “***C_a_***” is the added MO concentration, “***C_b_***” is the real MO concentration without adding the standard MO, and “***C_c_***” is the found MO concentration after adding standard MO [22].

## 3. Results and Discussion

### 3.1. Optimal Synthetic Conditions of AC-NCDs

To reach a high fluorescence property, different synthetic conditions including solvent volume percentages, hydrothermal temperatures, hydrothermal reaction time durations, and N-dopant weights were evaluated by comparing their fluorescence emission spectra. As for the percentages of solvent volume, the emission intensity was lower without adding ultrapure water to AC extract in the hydrothermal reaction process, while the emission intensity increased after adding different volume percentages of the solvent. The highest emission intensity was obtained with 75% of the solvent mixed with the AC fruit extract (Figure S1a). This result may be due to the chemical constituents in the AC fruit extract reacted slowly at a low solvent volume percentage. Furthermore, the temperature and reaction time duration of hydrothermal treatment possess significant effects on the fluorescence nature of CDs [23]. The AC-NCDs exhibited the highest emission intensity with a higher hydrothermal temperature and longer reaction time duration as depicted in Figure S1b,c. The emission intensity of AC-NCDs reached optimum intensity with the synthetic conditions of 15 h and 200 °C. The increase of temperature and the prolongation of hydrothermal reaction time duration can generate more carbon nucleus, exhibiting the increase of fluorescence properties. A lower hydrothermal temperature and shorter reaction time duration are inadequate conditions for the carbonization process, thus leading a lower emission intensity [24].

Furthermore, Arg weights added to the AC fruit extract solution were also checked (Figure S1d). The emission intensity of AC-NCDs was weak with a lower Arg weight (0.025 g). This result may be due to the small portion of N-source doped on the CDs. When the Arg weight was increased to 0.6 g, then the emission intensity increased and remained constant with 0.8 g Arg. The strong fluorescence properties of AC-NCDs may be generated from the emissive traps of the N-containing functional groups [25]. Therefore, the optimized hydrothermal synthetic conditions were 75% of solvent volume percentage, 200 °C of temperature, 15 h of reaction time duration, and 0.6 g of Arg weight. The appearances of synthetic products with 0.1 g Arg only (1, colorless), AC fruit extract solution only (2, bright brown), and AC fruit extract solution with different Arg weights (3–9, brown to dark brown) were shown in Figure S1e. The dark brown color indicated the highest production of AC-NCDs with the highest fluorescence properties. The emission intensity comparison of CDs from AC fruit extract solution, 0.1 g Arg, and AC fruit extract solution with 0.1 g Arg synthesized with the same hydrothermal conditions was displayed in Figure S2. The emission intensity of CDs was increased by the passivation of Arg as N-dopant to CDs.

**Figure 2:**
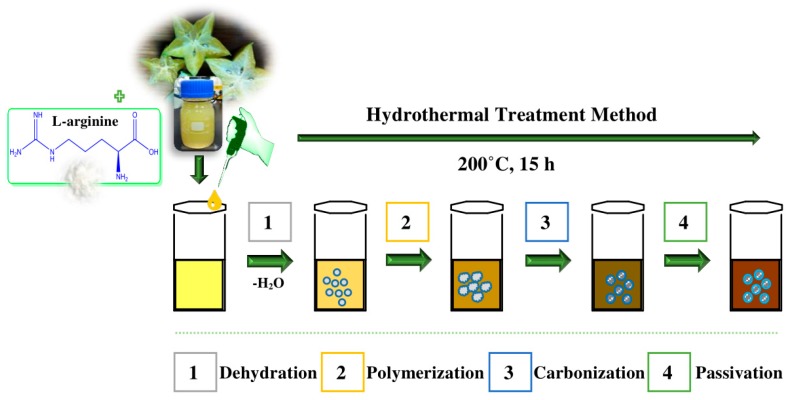
A proposed formation mechanism of AC-NCDs.

The formation mechanism of AC-NCDs is related to hydrothermal treatment conditions. The possible formation mechanism of AC-NCDs was illustrated in Figure 2. Several processes were involved in the formation of AC-NCDs during hydrothermal treatment including dehydration, polymerization, carbonization, and passivation [26,27,28,29]. Firstly, the –COOH, –C=O, –OH, and –NH_2_ groups presenting in AC fruit extract and Arg may react together, facilitating dehydration and carbonization process in elevated temperature. The continuous intermolecular dehydrationhappened during the hydrothermal process, inducing the shrinkage of polymer nanoparticles. The aromatic clusters were simultaneously produced inside the polymers and some C=C and C=N bonds form at this stage. The dehydration and polymerization will occur between the molecules through a well-known amidation reaction. Then, O atoms were substituted by N atoms to form amides inducing a newly formed surface state. A burst in the nucleation of NCDs takes place when the aromatic cluster concentration in the polymers reaches the critical supersaturation point. The resulting peptide chains were further carbonized with increasing NCDs particles through prolonged reaction time and then the polymeric fragments vanished. In the hydrothermal process, the surface passivation facilitates a high radiative recombination yield. As a result, a higher N content can increase the fluorescence QY and emission intensity of the CDs.

### 3.2. Characterizations of AC-NCDs

**Figure 3:**
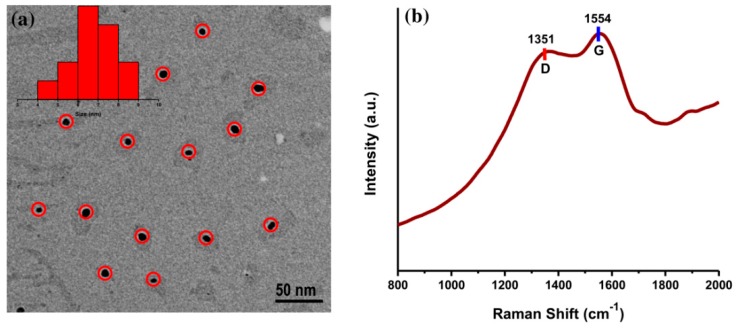
(**a**) Transmission electron microscopy (TEM) image and (**b**) Raman spectrum of AC-NCDs.

The surface morphology of AC-NCDs was captured by a TEM microscope. The TEM image of AC-NCDs with spherical shape and evenly distributed over the carbon-coated Cu grid was shown in Figure 3a. The size distribution of AC-NCDs was ranging from 4 to 9 with an average size of 6.67 nm. Besides, the Raman spectrum of AC-NCDs (Figure 3b) showed the disordered D band at ~1351 cm^−1^ and the crystalline G band at ~1554 cm^−1^ associated with the sp^3^- and sp^2^-carbon moieties in the AC-NCDs, respectively. The relative band intensity ratio (*I_D_/I_G_*) is a key parameter to predict the graphitization degree of carbon nanomaterials [30]. The *I_D_/I_G_* value of AC-NCDs was found to be 1.6 (in general, *I_D_/I_G_* is higher than 1 for well amorphous carbon), which indicates a low degree of graphitic order, sufficient major surface defects, and plenty of surface functional groups [31]. A high degree of defect formed by nitrogenation and oxygenation processes during synthesis partially destroys the graphitic structure of AC-NCDs [32,33]. Therefore, this Raman spectrum proved that the AC-NCDs have an amorphous structure and not graphene quantum dots.

**Figure 4:**
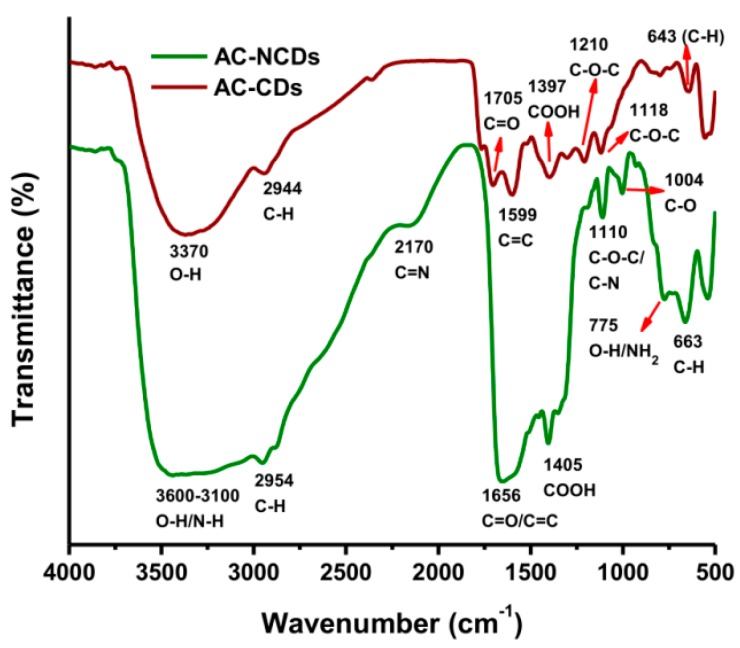
FTIR spectra of AC-CDs and AC-NCDs.

Furthermore, the functional groups of CDs were measured by FTIR spectroscopy. The FTIR spectra of AC-CDs (dark red line) and AC-NCDs (green line) were given in Figure 4. The FTIR spectrum of AC-CDs exhibited absorption bands at 3370, 2944, 1705, 1599, and 1397 cm^−1^, which conforms to the O–H, C–H, C=O, C=C, and –COOH stretching vibrations, respectively [22,34,35]. AC-CDs also exhibited the stretching vibration bands at 1210/1118 and 643 cm^−1^, corresponding to C–O–C and C–H bonds, respectively [36,37]. These functional groups were slightly shifted, decreased, and broadened in the FTIR spectrum of AC-NCDs due to the influence of carbonyl groups [37]. In the FTIR spectrum of AC-NCDs, the O–H stretching vibrations broadened ranging from 3600 to 3100 cm^−1^, which reveals that the CDs were doped by N-dopant from Arg [38]. The stretching vibrations of C–H groups shifted from 2944 to 2954 cm^−1^. The wide absorption bands of AC-NCDs centered at 1656 cm^−1^ were associated with C=O/C=C bonds [39,40]. The bands at 2170 and 1004 cm^−1^ were correlated to the stretching vibrations of C=N and C–O bonds, respectively [41,42]. The stretching vibrations of –COOH groups shifted from 1397 to 1405 cm^−1^. The band at 1110 cm^−1^ was identified as C–N and C–O–C stretching vibrations [38]. The other peaks at 775 and 663 cm^−1^ were identified as O–H stretching/NH_2_ wagging vibrations and C–H stretching vibrations, respectively [40,43].

**Figure 5:**
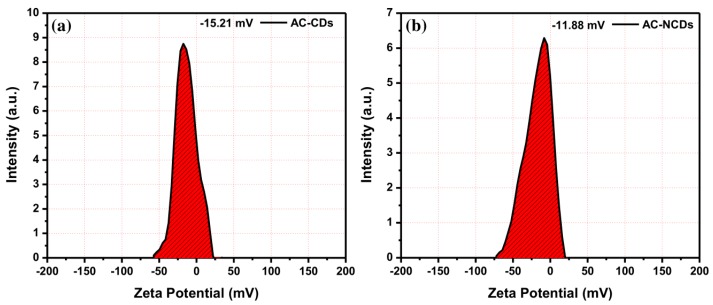
Zeta potential values of (**a**) AC-CDs and (**b**) AC-NCDs.

Moreover, to determine the electrical charges on the surface of nanoparticles and their colloidal stability, the zeta potential analysis was conducted. The zeta potential values of AC-CDs and AC-NCDs were measured and shown in Figure 5. The zeta potential value of AC-CDs was determined to be −15.21 mV (Figure 5a), which indicates the presence of negatively charged carboxyl (–COOH) and hydroxyl (–OH) functional groups on their surface. The zeta potential value of AC-NCDs was determined to be −11.88 mV (Figure 5b). The lowering of negatively charged from −15.21 mV to −11.88 mV was due to the presence of amine (–NH_2_) functional groups on the surface of AC-NCDs. This resulted in no amine groups being present on the surface of AC-CDs, making their zeta potential value more negative as a cumulative surface charge. The negative zeta potential values ensure good colloidal stability of AC-CDs and AC-NCDs.

**Figure 6:**
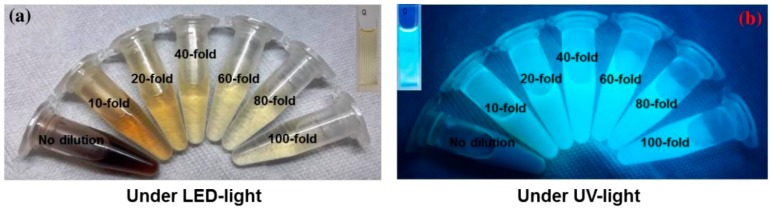
The appearance of AC-NCDs with various dilution factors under irradiation of (**a**) LED-light and (**b**) UV-light.

**Figure 7:**
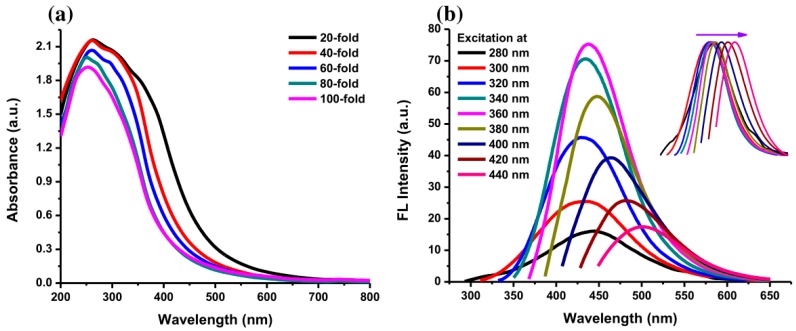
(**a**) UV-Vis absorption spectra of AC-NCDs with various dilution factors and (**b**) fluorescence emission spectra of AC-NCDs at excitation wavelengths of 280–440 nm with 20 nm increment (Inset: normalized fluorescence emission spectra).

The photograph in Figure 6a shows the color of AC-NCDs’ solution with different dilution factors (0 to 100-fold) under LED-light irradiation. The strong bright blue fluorescence color was emitted from the solution under UV-light irradiation of 365 nm at 20W power, which represents the formation of CDs (Figure 6b). AC-NCDs’ solution without dilution after synthesis did not emit bright blue fluorescence color because of a very high solution concentration while AC-NCDs with 60-fold of dilution showed strong bright blue fluorescence color confirmed with the optimum fluorescence emission intensity. This latter finding indicated that the AC-NCDs possessed suitable optical properties. Figure 7a represents the UV-vis absorbance of AC-NCDs with various dilution factors. The absorption peak behaviors are proportionate to the dilutions, while the peak centers were almost unchanged. The characteristic absorption peak at ~270 nm may be related to C=C bonds with π→π* transition [44]. The characteristic absorption peak located at ~325 nm is typically attributed to C=O bonds with n→π* transition [44]. The AC-NCDs exhibit strong UV absorption at the tail peak extending to the visible light region.

Furthermore, Figure 7b shows the fluorescence emission spectra of AC-NCDs with the excitation wavelengths of 280–440 nm. The optimum emission intensity was at 446 nm with 360 nm excitation after observing all fluorescence spectra. The 446 nm emission wavelength was associated with the highly bright blue fluorescence color of AC-NCDs. The emission intensity increased with increasing excitation wavelength from 280 to 360 nm and then decreased with the increase of the excitation wavelength from 380 to 440 nm. The clear excitation-dependent emission spectra are provided Inset Figure 7b. The emission peak centers red-shifted with increasing the excitation wavelengths, which may be attributed to various energy traps of AC-NCDs’ surface [45]. The effect of giant red-edge is the cause of the strong dependency of the emission peak center with different excitation wavelengths [46]. The QY of AC-NCDs was about 12.35% determined at 360 nm of excitation wavelength using quinine dissolved in sulfuric acid as the reference. A higher QY may be present because of the forming of good emissive states due to the presence of N-rich groups. Table S1 shows the comparison of synthetic methods, excitation wavelengths, emission wavelengths, and QYs of several NCDs [47,48,49,50,51]. The AC-NCDs have higher QY compared to other reported NCDs.

### 3.3. Fluorescence Stability of AC-NCDs

The fluorescence stability of AC-NCDs was investigated to examine the potential application of AC-NCDs as a fluorescent probe. The photostability study revealed that the light irradiation had no photo-bleaching effect on the emission intensity with continuous light irradiation until 1 h (Figure S3a). The photo-bleaching resistant nature of nanoparticles may be due to the negatively charged functional groups of AC-NCDs have the electrostatic repulsions each other, indicating their high photostability [52]. Further, the thermal stability on the fluorescence nature was checked by heating the AC-NCDs’ solution from low to high temperature (25–100 °C) in a sealed container. There was no significant decrease in the emission intensity even at 100 °C (Figure S3b), exhibiting their high thermal stability. A higher temperature does not cause the permanent devastation of the structure and functional groups of AC-NCDs [53]. The O- and N-functional groups may protect the nanoparticle aggregation of AC-NCDs at higher temperatures [53].

Furthermore, the effect of ionic strengths was also examined by the addition of various NaCl concentrations. AC-NCDs represented only a negligible decrease and a high resistance to salt concentrations up to 1000 mM, which further expands their application to identical ionic-rich environments (Figure S3c). The salt ionic strength can effectively control the aggregation of AC-NCDs and AC-NCDs particles can individually separate under salt disturbances, showing a homogeneous phase without precipitation [50]. Moreover, the shelf-life of AC-NCDs was examined by keeping them at room temperature. After storing for 30 days, no significant alterations were monitored in the emission intensity, indicating their high stability and longer shelf-life (Figure S3d). The AC-NCDs’ solution shows a homogeneous phase in the long-term without clear precipitation.

Also, fluorescence measurements of AC-NCDs were performed with various pH values by adapting the pH values with NaOH and HCl solutions (0.1 M). It can be observed that the fluorescence emission intensity changed by changing the pH value and the highest emission intensity was obtained with pH 5 (Figure S3e). The pH 2 decreased the emission intensity because of the saturated protonation of AC-NCDs in a strong acid condition [54]. The increase of emission intensity was observed after increasing the pH value from 2 to 5, and then the emission intensity decreased gradually by shifting the pH value from 5 to 12. These characteristics could be associated with the distinct deprotonation degrees of AC-NCDs at various pH values [55]. The AC-NCDs have pH-dependent fluorescent nature because they serve as electron donors or acceptors. H^+^ or OH^-^ ions may prevent the electron transfer process which induces the significant change of functional groups [56]. The AC-NCDs show a good fluorescent character in a weak acid condition, while the comparatively low fluorescent characters were shown in strong acid, neutral and alkaline conditions.

### 3.4. Fluorescent Probe of MO

**Figure 8:**
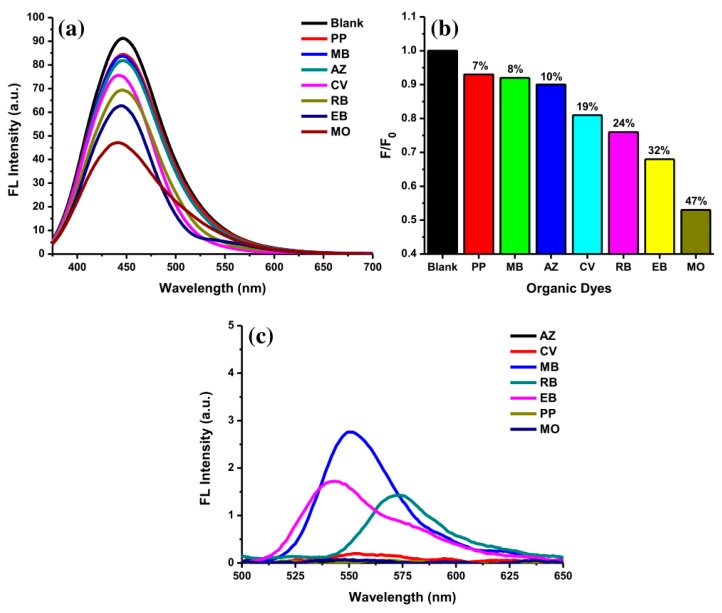
(**a**) Fluorescence emission spectra of AC-NCDs’ solution with various ODs (50 μM), (**b**) fluorescence quenching percentages of AC-NCDs’ solution in the existence of various ODs, and (**c**) fluorescence emission spectra of ODs (50 μM) at 360 nm excitation wavelength.

The selectivity of AC-NCDs for several ODs was evaluated at pH 5. Figure 8a showed fluorescence spectra of AC-NCDs after adding with several ODs including PP, MB, AZ, CV, RB, EB, and MO (50 μM). The fluorescence emission peak of AC-NCDs was centered on a specific wavelength after interacting with the ODs. The emission peak center of AC-NCDs at 446 nm was maintained after interacting with PP, MB, AZ, and RB. The emission peak center of AC-NCDs was shifted from 446 nm to 444 nm, 441 nm, and 440 nm after interacting with EB, CV, and MO, respectively. The largest shift of emission peak center was occurred by MO. Therefore, this characteristic is also one of the marks for the MO interaction to AC-NCDs. Almost all ODs quenched the fluorescence intensity of AC-NCDs with different quenching percentages. Figure 8b shows the fluorescence quenching efficiency for MO (47%) which is higher than EB (32%), RB (24%), CV(19%), AZ (10%), MB (8%), and PP (7%), leading to the highest selectivity of AC-NCDs for MO. The individual fluorescence emission peak of ODs without interacting with AC-NCDs was also recorded at the excitation wavelength of 360 nm (Figure 8c). As compared to other ODs, only MB, EB, and RB showed their very weak emission peaks with the emission wavelength of 550, 545, and 470 nm, respectively. Because of very weak emission peaks derived from MB, EB, and RB, and no emission peaks derived from PP, CV, AZ, and MO, so that there was no other fluorescence peak showed after interacting ODs with AC-NCDs except at 440 nm, which corresponds to AC-NCDs’ character. So, the fluorescence quenching of AC-NCDs after interacting with MO is the most important behavior for probing MO.

**Figure 9:**
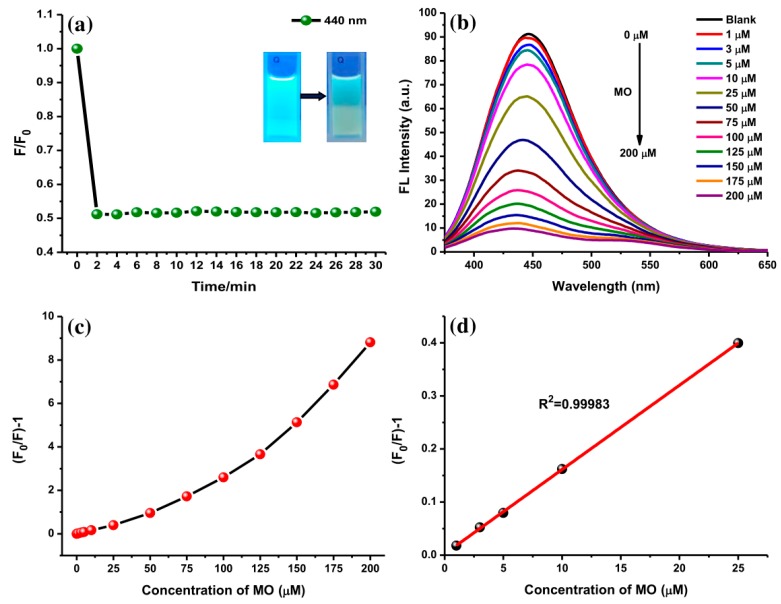
(**a**) Incubation time for the AC-NCDs/MO interaction on the fluorescence behavior (Inset: AC-NCDs’ and AC-NCDs/MO’ solutions under UV-light irradiation), (**b**) fluorescence emission spectra of AC-NCDs’ solution in the existence of various MO concentrations (360 nm excitation wavelength 360 nm, 3 min interaction time), (**c**) plots of (F_0_/F)-1 values of AC-NCDs’ solution in the presence of various MO concentrations (0–200 μM), and (**d**) the relationship between the fluorescence quenching and MO concentrations of 1 to 25 μM.

Furthermore, the interaction of AC-NCDs and MO with different concentrations was studied to explore their sensitivity on the fluorescence emission intensity. The kinetic experiments disclosed that within 3 min of interaction (1 min of mixing time, 2 min of incubation time), the emission intensity of AC-NCDs quenched to a fixed value which did not alter even after 30 min (Figure 9a). This result indicates that the reaction between AC-NCDs and MO was faster and more stable, recommending expectant applicability for rapid detection of MO without any tight time control. The MO reduced the fluorescence properties of AC-NCDs under UV-light irradiation by dimming the bright blue color as shown Inset Figure 9a. The sensitivity of AC-NCDs was assessed by measuring their fluorescence emission intensities toward various MO concentrations (1–200 μM). As displayed in Figure 9b, the emission intensity of AC-NCDs gradually quenched with the increase of MO concentration. The gradual quenching of emission intensity exhibited a linear response, (F_0_/F)-1, to the MO concentrations, where F_0_ and F refer to the fluorescence emission intensities at 446 nm without and with MO addition, respectively (Figure 9c). The calibration curve of quenching efficiency depicted as (F_0_/F)-1 with the MO concentrations ranging from 1 to 25 μM has a good correlation coefficient (R^2^) of 0.99983 with 0.30 μM detection limit as represented in Figure 9d.

In recent years, several detection methods for MO have been performed to improve the detection sensitivity. These methods include a Micellar Liquid Chromatography [18], an electrochemical sensor based on Smectite-HDTMA/GCE [19], a SERS probe based on *β*-CD@Ag NP monolayer [20], and an extraction by using chitosan-zinc oxide NPs [21]. In Table S2, the results of linear ranges and detection limits from the reported methods and this method for probing MO were compared. The proposed AC-NCDs’ fluorescent probe offered good sensitivity with wider linearity and a relatively lower limit of detection. This method can be a preference for the MO detection in aqueous solution. Almost all of the reported methods need specific equipment, a sophisticated technique, or complex operations. Conversely, this fluorescent probe has some benefits including cheap instrumentation, easy in operation, and quick response making it more usable for routine MO detection in environments. The prepared AC-NCDs’ solution (10 mL) from one-time of synthesis can be used for more than 600 times of measurements. For each measurement, only 15 µL of AC-NCDs’ solution was required. So, this is also one of the important benefits of using AC-NCDs as a fluorescent probe.

To further assess the applicability and feasibility of this method, the AC-NCDs as a fluorescent probe was utilized to determine the MO trace level in tap water samples. Because no MO was detected in the tap water samples, the samples were separately spiked with five MO concentration levels and the quantification results are summarized in Table S3. Three measurements were carried out for each concentration. The recovery measurements were carried out to assess the method’s accuracy. It can be seen that the recovery results of MO in these spiked samples were in the range of 99.27 to 99.79% with <1% RSDs. The MO concentrations determined by the AC-NCDs’ fluorescent probe are very close to the concentrations obtained in ultrapure water. This evidence reveals the applicability of the proposed fluorescent probe for accurate detection of MO in real water samples. Therefore, the results confirmed that the AC-NCDs with their accuracy, sensitivity, and reliability can be potentially utilized to detect MO in environmental water samples.

### 3.5. The pH Effect on Interaction and Quenching Mechanisms

**Figure 10:**
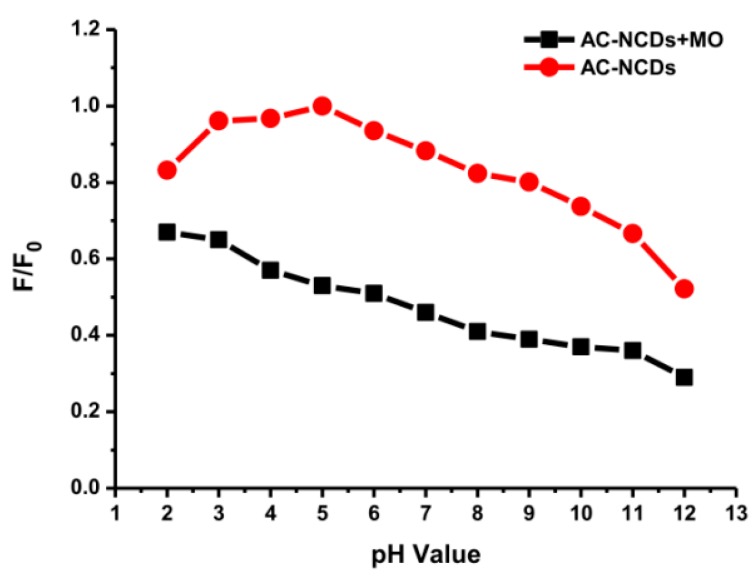
Fluorescence intensity of AC-NCDs and AC-NCDs/MO system with different pH values.

**Figure 11:**
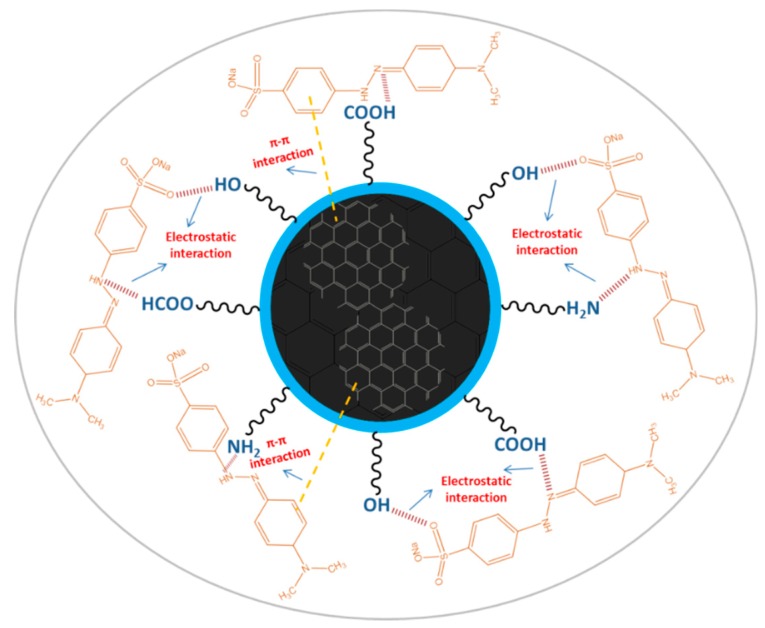
A schematic illustration representing plausible interactions between the AC-NCDs and MO.

The pH values present a significant role in the interaction process, influencing not only the surface charge of AC-NCDs and dissociation of surface functional groups of AC-NCDs but also the chemical speciation of MO molecules [57]. The effect of pH values from 2 to 12 on the interaction of AC-NCDs and MO to investigate further quenching efficiency was shown in Figure 10. The optimal interaction was achieved at pH 5 due to the highest quenching efficiency of AC-NCDs after interacting with MO due to electrostatic interaction [58]. The lower interaction of AC-NCDs and MO was because of H^+^ and OH^-^ interruptions in strong acid and alkaline conditions. MO has two different chemical structures with quinone or azo bond chromophores, depending on the solution pH [59]. MO occurs as a quinone form in acid conditions and converts into an azo structure in alkaline conditions [60]. The MO quinone form has two major active positions, –S=O and –NH– groups. The surface of AC-NCDs has low positive or low negative charge under acidic conditions (Figure S4), increasing the electrostatic interaction to the –S=O groups and/or to the –NH– groups of MO. In the –S=O groups the hydrogen bonding acceptor interacts with –OH groups on the surface of AC-NCDs, while in the –NH– groups of MO the hydrogen bonding donor interacts with the –COOH groups or –NH_2_ groups on AC-NCDs. Besides, π–π interactions between C=C or benzene rings of MO in both azo and quinone type structures and bulk π-systems of AC-NCDs also play a slight role in the interaction [61]. A schematic of the plausible interactions between AC-NCDs and MO is illustrated in Figure 11.

The interaction of AC-NCDs and MO was reduced significantly at the pH value lower than 5 which may result from slow interaction in strong acid conditions with excess H^+^ ions, which interrupt the electron transfer from AC-NCDs to MO. The interaction between AC-NCDs and MO increased significantly at pH 5 and slightly reduced to pH 9. It can be illustrated that the quenching efficiency increased obviously with the increase of pH ranging from pH 2 to pH 5 (16% to 47%) and slightly decrease to pH 9 (41%) and decrease further to pH 12 (23%). The increase of solution pH value provides that the AC-NCDs’ surface has a higher negative charge and electrostatic repulsion with MO, resulting in a decreased percentage of their interaction. The excess of OH^-^ ions in strongly alkaline conditions contributes to the surface charge of AC-NCDs which showed a higher negative zeta potential value (Figure S4). Also, the MO azo form has only one main active position, –S=O groups. As a result, the fewer amount of hydrogen bonding is formed when MO is shifted from quinone form to azo form, which results in a decrease of interaction capacity [62]. Therefore, the fluorescent probe can be performed in the pH range from 5 to 9 where the fluorescence quenching efficiency is still in a tolerable range “47%–41%”. Considering its superior detection performance, pH 5 was the most suitable and, therefore, would be used as an expectant pH value for the streamlined detection of MO because of higher fluorescence emission intensity of AC-NCDs and higher interaction with MO at this pH value.

Other possible quenching mechanisms were investigated by comparing the absorbance of MO and the fluorescence emission peak of AC-NCDs. As depicted in Figure S5a, MO showed two UV-vis absorption peaks at 270 nm and 460 nm while the AC-NCDs exhibited the highest emission intensity at 446 nm. Because a great spectral overlap between the emission wavelength of the fluorescent agent/excited electrons (AC-NCDs-energy donor) and the absorption spectrum of the quencher/electron-deficient molecules (MO-energy acceptor) is necessary for the inner filter effect (IFE) and energy transfer, so the fluorescence quenching of AC-NCDs is possibly due to the IFE and energy transfer (Figure S5a) [63,64]. The IFE happens upon the absorption of emitted light by the absorber. Thus, the IFE and energy transfer efficiency depends on the spectral overlap between the absorber (energy acceptor) and emitter (energy transfer). According to energy transfer efficiency equation, E = 1 − (F_D_/F_D_′) [65] (where E = energy transfer efficiency of the system, F_D_ = the integrated fluorescence intensity of the donor with the presence of acceptor, F_D_′ = the integrated fluorescence intensity of the donor with the absence of acceptor), the maximum energy transfer efficiency was calculated to be 44.0% for 50 µM MO (Figure S6a) and 66.8% for 100 µM MO (Figure S6b). To achieve the pH-dependent effect of MO on AC-NCDs, the UV-vis absorption of the AC-NCDs/MO system at various pH values was evaluated. The increasing of fluorescence quenching efficiency could be related to the MO-induced alterations of the absorption peak. As represented in Figure S5b, the MO absorbance at 460 nm slightly reduced when the AC-NCDs/MO system at pH 5; thus, the quenching efficiency of AC-NCDs was higher than pH 12 with additional lower absorbance at 460 nm. The quenching efficiency of the AC-NCDs/MO system at pH 5 and pH 12 was still higher than that at pH 2 as confirmed with the fluorescence quenching percentages. From the normalized absorption spectra, these pH values (5 and 12) produced the spectral overlaps between the absorption peak of MO and the emission spectrum of AC-NCDs. The spectral overlap reduction appeared at pH 2 by shifting the wavelength from 460 nm to 505 nm with changing its color from yellow to red as shown in Figure S7, indicating the quenching reduction based on the decrease of IFE and energy transfer effects. The results denoted that the IFE and energy transfer efficiency of AC-NCDs by MO was pH-dependence.

## 4. Conclusions

A rapid and facile fluorescent probe-based on AC-NCDs was successfully utilized for MO detection. The AC-NCDs were prepared through a simple and green hydrothermal treatment method using *A. carambola* fruit extract and Arg as a carbon precursor and a nitrogen dopant for the first time, respectively. The AC-NCDs were spherical with 6.67 nm of average size. The optimum fluorescence emission intensity was centered at 446 nm with 360 nm excitation wavelength. The fluorescent quantum yield was calculated to be 12.35%. The AC-NCDs were used as a fluorescent probe for MO with higher selectivity and sensitivity. The quenching percentages were affected by the pH values of the system. The plausible interaction mechanisms were driven by electrostatic interaction, π–π interaction, inner filter effect, and energy transfer. Under optimum conditions, the calibration curve of the fluorescent probe was linear from 1 to 25 μM range with R^2^ of 0.99983 and a 0.30 μM detection limit. This work provides a potential application of AC-NCDs to detect MO for environmental protection.

## Supplementary Materials

The following data are available online at https://www.mdpi.com/1424-8220/19/22/5008/s1, Figure S1: Fluorescence intensity of AC-NCDs prepared at different (a) solvent volume percentages, (b) hydrothermal temperatures, (c) reaction time durations, (d) weights of Arg in 10 mL AC fruit extract solutions, and (e) the appearances of synthetic products (1: 0.1 g L-arginine only in 10 mL H_2_O, 2: AC fruit extract solution only, 3–9: AC fruit extract solution with L-arginine (0.025, 0.05, 0.1, 0.2, 0.4, 0.6, 0.8 g, respectively)). Synthetic conditions were kept at 200 °C of temperature and 15 h of reaction time duration, Figure S2: Fluorescence intensity of (a) CDs from AC fruit extract solution, (b) CDs from 0.1 g Arg in 10 mL ultrapure water, and (c) AC-NCDs prepared from AC fruit extract solution with 0.1 g Arg. The excitation wavelength was 360 nm and the synthetic conditions were kept at 200 °C for 15 h, Figure S3: The fluorescence spectra comparison of AC-NCDs’ solution with various (a) light irradiation times, (b) heating temperatures, (c) salt concentrations, (d) storage times, and (e) pH values, Figure S4: Zeta potential graphs of AC-NCDs’ solutions at different pH values (2, 5, 9, and 12), Figure S5: (a) The normalized fluorescence emission spectrum of AC-NCDs and UV-vis absorption spectrum of MO, and (b) UV-vis spectra comparison of AC-NCDs, MO, and AC-NCDs/MO system (pH 2, 5, and 12). The MO concentration was 50 μM, Figure S6: FRET measurement according to the equation E = 1 − (F_D_/F_D_’). The excitation wavelength was 360 nm; the integrated fluorescence intensity was calculated from 360 nm to 650 nm with (a) 50 µM MO and (b) 100 µM MO, Figure S7: The appearance of AC-NCDs/MO’ solution with different pH values (MO concentration was 50 μM), Table S1: The comparison of starting materials, synthetic methods, excitation wavelengths (λ_ex_), emission wavelengths (λ_em_), and quantum yield (QY) from some N-doped CDs, Table S2: The comparison of this method with other methods for the probing of MO, Table S3: The probing of MO in tap water samples (n = 3).
