# Supplementary material for: Nitrogen-Doped Carbon Dots from Averrhoa carambola Fruit Extract as a Fluorescent Probe for Methyl Orange
Source: Sensors (Basel). 2019 Nov 16;19(22):5008. doi: 10.3390/s19225008 (PMC6891795; doi:10.3390/s19225008)
Supplement: Supplementary file 1 [file sensors-19-05008-s001.pdf]

Electronic Supporting Information

Nitrogen-Doped Carbon Dots from *Averrhoa carambola* Fruit Extract as a Fluorescent Probe for Methyl Orange

Muhammad Zulfajri, Sandhiya Dayalan, Wang-Yu Li, Chia-Jung Chang,[1] Yuan-Pin Chang, and Genin Gary Huang

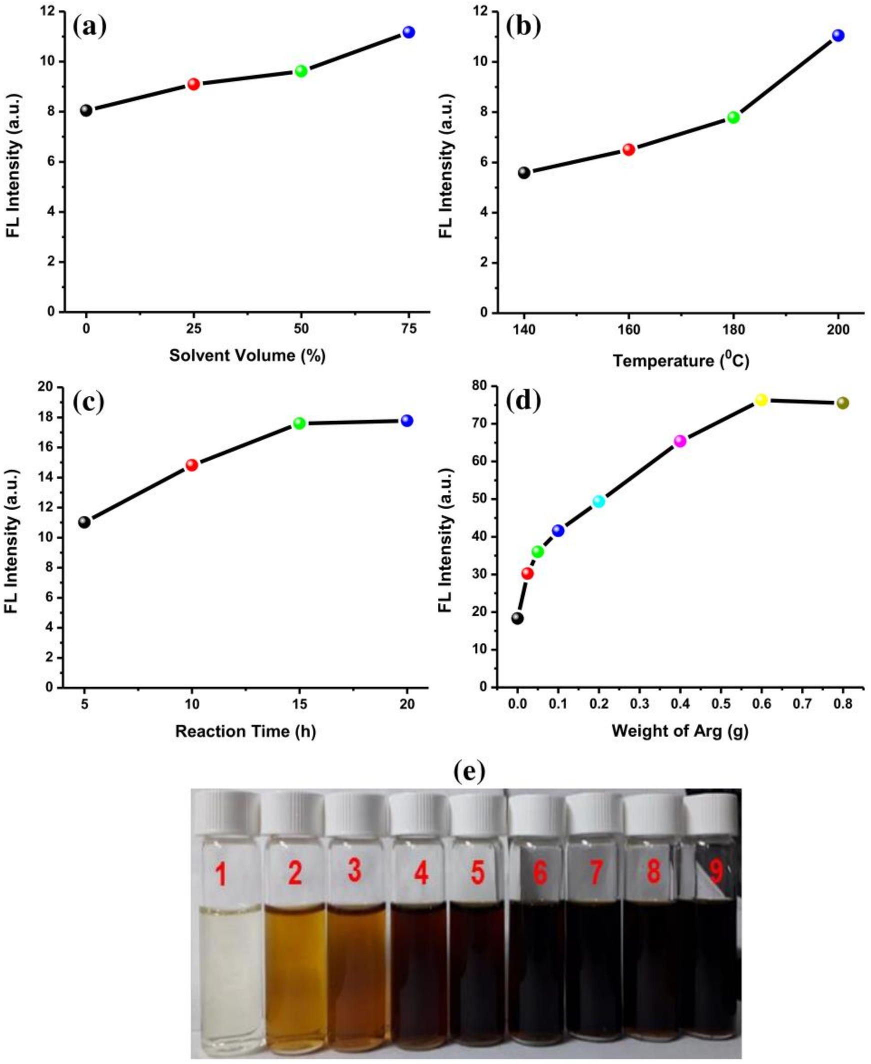

Figure S1. Fluorescence intensity of AC-NCDs prepared at different **(a)** solvent volume percentages, **(b)** hydrothermal temperatures, **(c)** reaction time durations, **(d)** weights of Arg in 10 mL AC fruit extract solutions, and (**e**) the appearances of synthetic products (1: 0.1 g L-arginine only in 10 mL H_2O, 2: AC fruit extract solution only, 3-9: AC fruit extract solution with L-arginine (0.025, 0.05, 0.1, 0.2, 0.4, 0.6, 0.8 g, respectively)). Synthetic conditions were kept at 200˚C of temperature and 15 h of reaction time duration.

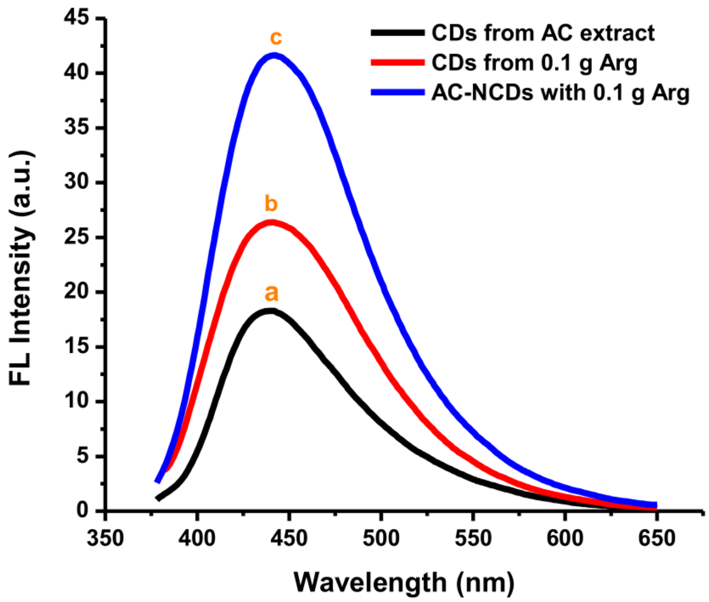

Figure S2. Fluorescence intensity of (a) CDs from AC fruit extract solution, (b) CDs from 0.1 g Arg in 10 mL ultrapure water, and (c) AC-NCDs prepared from AC fruit extract solution with 0.1 g Arg. The excitation wavelength was 360 nm and the synthetic conditions were kept at 200˚C for 15 h.

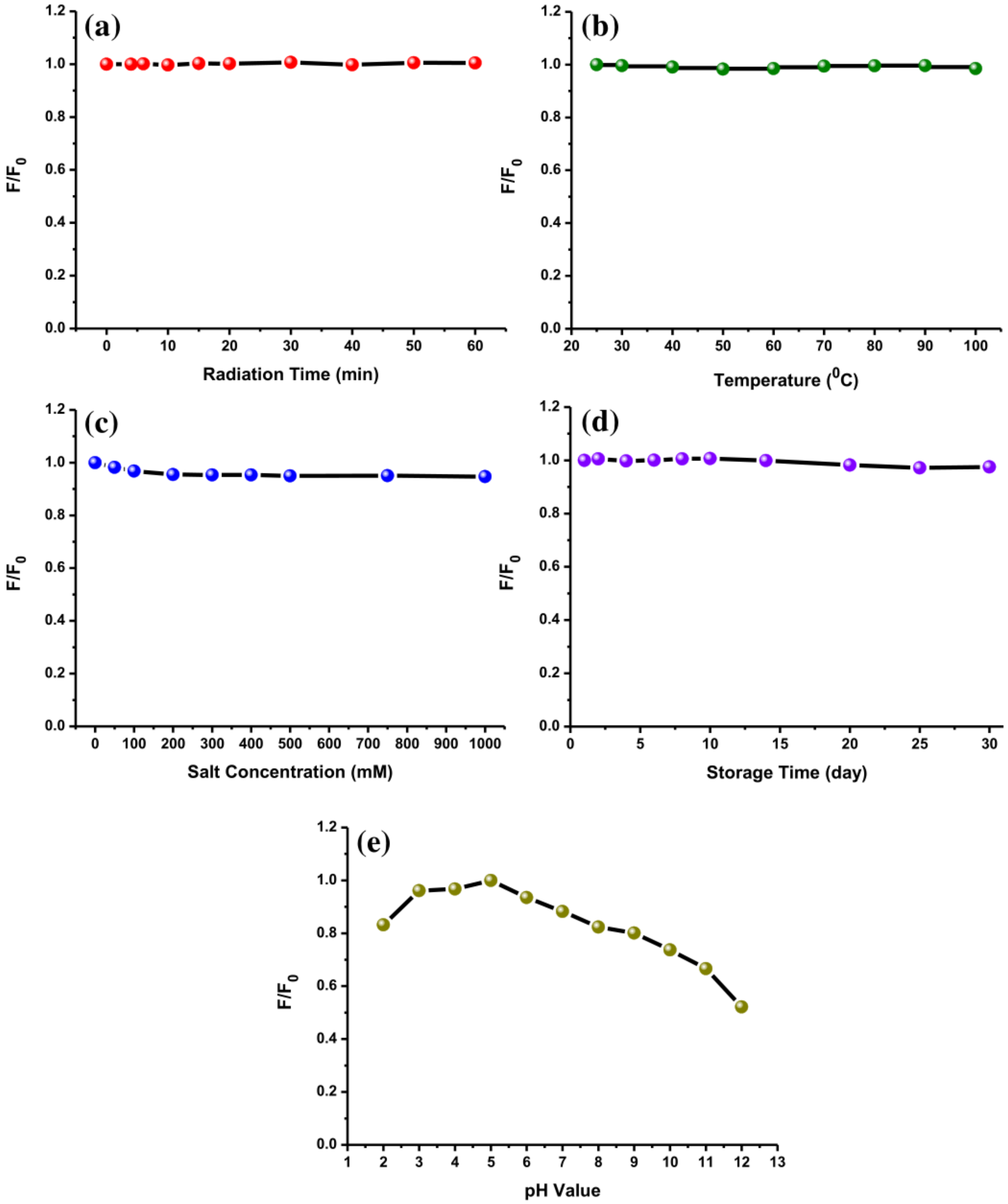

Figure S3. The fluorescence spectra comparison of AC-NCDs' solution with various (a) light irradiation times, (b) heating temperatures, (c) salt concentrations, (d) storage times, and (e) pH values.

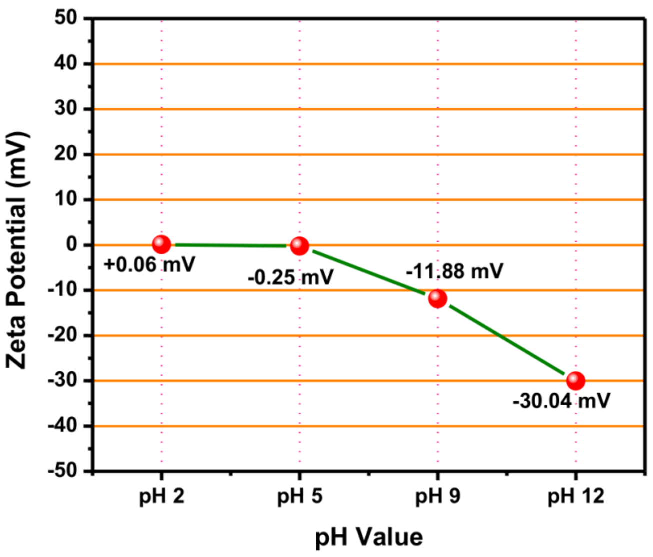

Figure S4. Zeta potential graphs of AC-NCDs' solutions at different pH values (2, 5, 9, and 12).

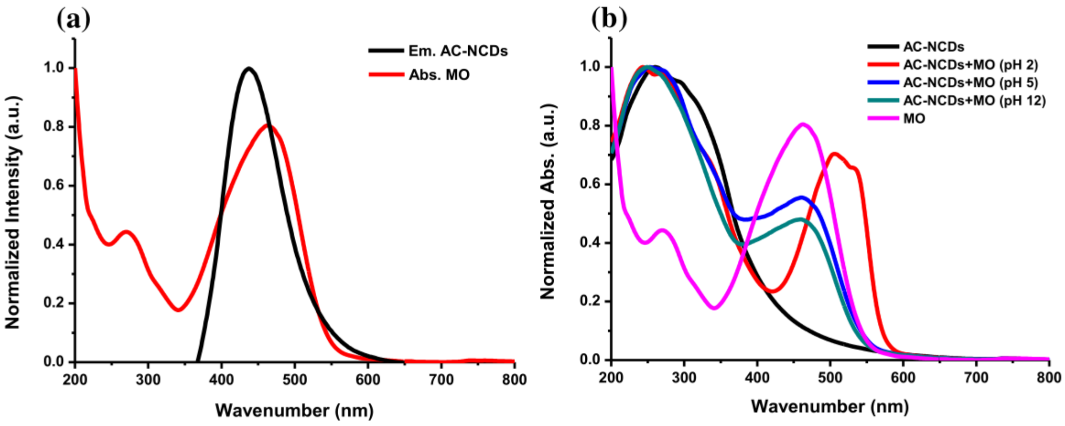

Figure S5. (a) The normalized fluorescence emission spectrum of AC-NCDs and UV-vis absorption spectrum of MO dye, and (b) UV-vis spectra comparison of AC-NCDs, MO, and AC-NCDs/MO system (pH 2, 5, and 12). The MO concentration was 50 μM.

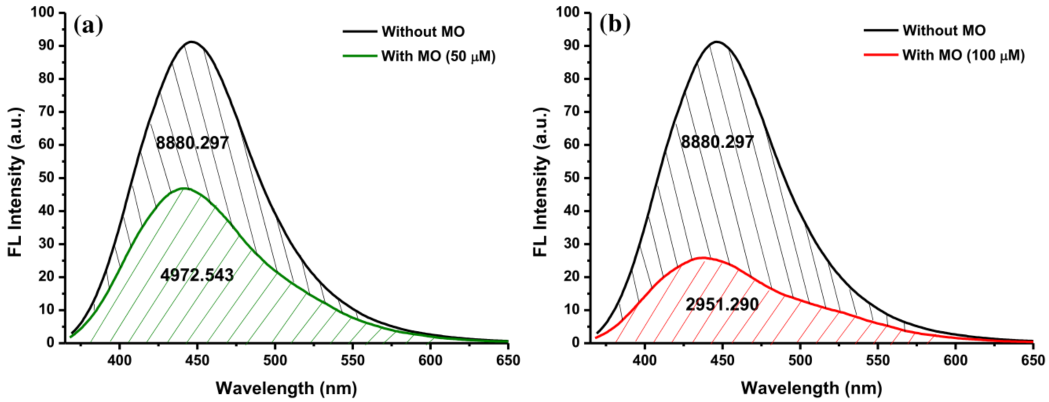

Figure S6. FRET measurement according to the question E=1-(FD/FD'). The excitation wavelength was 360 nm; the integrated fluorescence intensity was calculated from 360 nm to 650 nm with (a) 50 μM MO and (b) 100 μM MO.

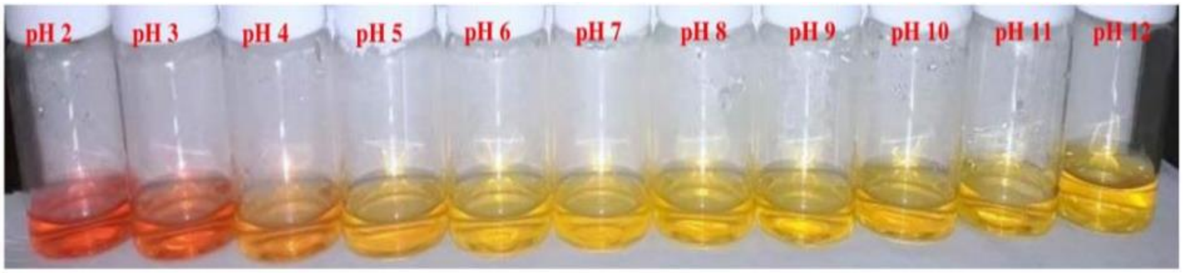

Figure S7. The appearance of AC-NCDs/MO system with different pH values (MO concentration was 50 μM).

Table S1.The comparison of starting materials, synthetic methods, excitation wavelengths (λex), emission wavelengths (λem), and quantum yield (QY) from some N-doped CDs

No.	Starting Material	Method	λ_{ex} (nm)	λ_{em} (nm)	QY (%)	Ref.
1	Glucose + *m*-phenylenediamine	Microwave	370	500	11.2	48
2	*Citric acid* + 1,10-Phenanthroline	Solid State	360	440	10	49
3	*Chionanthus retusus fruit* + Ammonia	Hydrothermal	340	425	9	50
4	Lemon fruit + L-arginine	Hydrothermal	340	490	7.7	51
5	*Prunus avium* fruit + Ammonia	Hydrothermal	310	411	13	52
6	AC fruit + L-arginine	Hydrothermal	360	440	12.35	This work

Table S2. The comparison of this method with other methods for the probing of MO

No	Detection method/based on	Linear range (μM)	LOD (μM)	Ref.
1	Micellar Liquid Chromatography	0.15-15	0.15	18
2	Electrochemical/Smectite-HDTMA/GCE	0.1-1.6	0.04	19
3	SERS/β-CD@Ag NP monolayer	0.5-10	0.50	20
4	Extraction/Chitosan-zinc oxide NPs	10-1000	0.70	21
5	Fluorescent probe/AC-NCDs	1-25	0.30	This work

Table S3. The probing of MO in tap water samples (n = 3).

No.	Added (μM)	Found (μM)	R (%)	RSD (%)
1	0	ND[a]	-	-
2	5	4.98 ± 0.01	99.59	0.25
3	10	9.97 ± 0.03	99.68	0.34
4	20	19.96 ± 0.02	99.79	0.09
5	50	49.63 ± 0.08	99.27	0.21
6	75	74.78 ± 0.11	99.71	0.18

[a]ND: not detected
